# Prognostic Factors Influencing Infectious Complications after Cytoreductive Surgery and HIPEC: Results from a Tertiary Referral Center

**DOI:** 10.1155/2019/2824073

**Published:** 2019-05-02

**Authors:** Maurizio Cardi, Simone Sibio, Francesco Di Marzo, Francesco Lefoche, Claudia d'Agostino, Giovanni Battista Fonsi, Giuseppe La Torre, Ludovica Carbonari, Paolo Sammartino

**Affiliations:** ^1^Dipartimento di Chirurgia Pietro Valdoni, Sapienza Università di Roma, Viale del Policlinico 155, 00161 Roma, Italy; ^2^Chirurgia Generale, Ospedale Versilia, Via Aurelia 335, 55041 Lido di Camaiore, Italy; ^3^Dipartimento di Malattie Infettive e Tropicali, Sapienza Università di Roma, Viale del Policlinico 155, 00161 Roma, Italy; ^4^Dipartimento di Sanità Pubblica e Malattie Infettive, Sapienza Università di Roma, Viale del Policlinico 155, 00161 Roma, Italy

## Abstract

**Background:**

Cytoreductive surgery (CRS) and hyperthermic intraperitoneal chemotherapy (HIPEC) showed promising results in selected patients. High morbidity restrains its wide application. The aim of this study was to report postoperative infectious complications and investigate possible correlations with the preoperative nutritional status and other prognostic factors in patients with peritoneal metastases treated with CRS and HIPEC.

**Methods:**

For the study, we reviewed the clinical records of all patients with peritoneal metastases from different primary cancers treated with CRS and HIPEC in our Institution from November 2000 to December 2017. Patients were divided according to their nutritional status (SGA) into groups A (well-nourished) and B/C (mild or severely malnourished, respectively). Possible statistical correlations between risk factors and postoperative complication rates have been investigated by univariate and multivariate analysis.

**Results:**

Two hundred patients were selected and underwent CRS and HIPEC during the study period. Postoperative complications occurred in 44% of the patients, 35.3% in SGA-A patients, and 53% in SGA-B/C patients. Cause of complications was infective in 42, noninfective in 37, and HIPEC related in 9 patients. Infectious complications occurred more frequently in SGA-B/C patients (32.6% vs. 9.8% of SGA-A patients). The most frequent sites of infection were surgical site infections (SSI, 35.7%) and central line-associated bloodstream infections (CLABSI, 26.2%). The most frequent isolated species was Candida (22.8%). ASA score, blood loss, performance status, PCI, large bowel resection, postoperative serum albumin levels, and nutritional status correlated with higher risk for postoperative infectious complications.

**Conclusions:**

Malnourished patients undergoing cytoreductive surgery and hyperthermic intraperitoneal chemotherapy are more prone to postoperative infectious complications, and adequate perioperative nutritional support should be considered, including immune-enhancing nutrition. Sequential monitoring of common sites of infection, antifungal prevention of candidiasis, and careful patient selection should be implemented to reduce the complication rate.

## 1. Introduction

Peritoneal metastases have long been considered a terminal event in the natural history of many gynecological and gastrointestinal (GI) cancers [1, 2], and the role of surgery for patients with peritoneal surface malignancies has been mostly palliation for tumor-related complications. Since the introduction of cytoreductive surgery (CRS) and hyperthermic intraperitoneal chemotherapy (HIPEC) by Sugarbaker in the early 90s [3], this treatment has expanded [4–13]. Despite promising results, these complex surgical procedures carry high rates of postoperative complications and mortality, restraining the wide diffusion and feasibility of these techniques [10, 11, 14–16].

A number of studies extensively report surgical and HIPEC-related complications, but only few of them are focused on postoperative infectious complications [2, 17–20]. Moreover, nutritional status has been shown to have a great impact on the immune system making patients with impaired host immune response more susceptible to postoperative complications after gastrointestinal surgery [21–24] including patients with ovarian and advanced colorectal cancer [25, 26], but the literature still lacks data about the impact of nutritional status in the outcome of patients treated with CRS and HIPEC for peritoneal carcinomatosis.

This is why we critically reviewed our Institution's clinical database of the patients who underwent CRS and HIPEC for peritoneal metastases with the aim to investigate correlations between possible prognostic factors and postoperative infectious complications, focusing on nutritional status and type of surgical procedures.

## 2. Materials and Methods

Patients with the diagnosis of peritoneal metastases from different primary cancers treated with cytoreductive surgery and HIPEC were selected from a prospectively maintained database in our Institution during a 17-year period from November 2000 to December 2017. Inclusion criteria were as follows: confirmed peritoneal metastases (histologically and/or cytologically); age < 75 years; ECOG performance status 0-2 [27]; adequate renal, liver, and bone marrow function; resectable disease; and preoperative serum albumin level > 2 g/dl. All patients had a Body Mass Index (BMI) between 18.50 and 24.99 kg/m^2^, a range that defines the normal weight population. We excluded from the study patients with extra abdominal metastases and/or unresectable disease. Nutritional status was evaluated as a risk factor for the development of infectious complications and assessed by providing each patient with a Subjective Global Assessment (SGA) form [17]. Patients were classified into 3 groups: well nourished (A), mildly moderately malnourished (B), and severely malnourished (C). BMI, albumin level, and cholesterol as well as triglycerides levels were considered to assess the need for Total Parenteral Nutrition (TPN) support prior to surgery. Mildly (B) or severely (C) malnourished patients received a minimum of 15 days of TPN and vitamin supplementation.

All patients received antibiotic prophylaxis at induction of anesthesia, with 2 g iv of ceftriaxone and 500 mg iv of metronidazole, repeated every 4 hours during the procedure and 24 hours after surgery.

Surgical procedures and HIPEC techniques have been described in previous reports [3, 10]. The extent of peritoneal disease and results of cytoreduction were classified using the peritoneal cancer index (PCI) and completeness of cytoreduction score (CC score) according to Sugarbaker [28]. According to protocols in use in our Institution, the drug used for HIPEC in 112 patients with gynecologic (ovarian, endometrial, or breast) primary cancers was cisplatin (75 mg/m^2^), while in 79 patients with colorectal, gastric, and pseudomyxoma peritonei, the drug was oxaliplatin (460 mg/m^2^ in 2 l/m^2^ of dextrose solution) plus intravenous infusion of 5-FU (400 mg/m^2^). The 9 cases of mesothelioma underwent cisplatin and doxorubicin. Patients were sent to the intensive care unit (ICU) in the immediate postoperative period for at least 24 hours.

In all symptomatic patients with suspected infection (fever > 38°C, dyspnea, dysuria, purulent surgical wound or drain, increasing white cell count, and C-reactive protein-CRP levels), blood cultures (a total of 3 samples, taken every 30 min from both central venous lines (CVLs) and the peripheral vein) and culture of the CVL tip and of biological fluids including those from abdominal drainage and urine cultures were performed. In case of sepsis from intra-abdominal infection, an abdominal CT scan or ultrasound was carried out to rule out and eventually to drain an intra-abdominal fluid collection.

An empiric antimicrobial therapy with carbapenem and teicoplanin was started until microbiologic results were obtained. Antifungal therapy was associated when a fungal infection was also suspected. Infected CVLs were removed and replaced 48/72 hours after the beginning of the therapy. Removal of the bladder catheter was mandatory after Candida contamination was proven. Once infection resolved, antimicrobial drug washout was performed for at least 24 hours, followed by blood cultures and urinalysis. No further investigations were carried out in the case of symptom-free patients.

For each patient, we recorded demographic, clinical, and pathological data: age, sex, comorbidities, nutritional status assessment, type of primary tumor, ECOG performance status, ASA score, time of cytoreduction (primary or secondary), preoperative chemotherapy, type and length of surgical procedures, PCI and CC score, blood loss, length of ICU, and overall postoperative stay. Observed postoperative adverse events have been recorded and divided as infectious, surgical, or HIPEC-related and graded according to the National Cancer Institute Common Terminology Criteria for Adverse Events (CTCAE) 5.0 [29]. The WHO chemotherapy toxicity scale was used to score cisplatin toxicity [30]. In the case of infectious complications, site of infection, type of sample collected, and etiology of the infection were recorded.

Possible statistical correlations between risk factors and postoperative complications rates have been investigated by the mean of univariate analysis (log-rank test). Results were matched by the mean of multivariate analysis (Cox regression model) to identify independent variables influencing infections occurring, using the Statistical Package for the Social Sciences for Windows (SPSS GmbH, Munich, Germany). The 2-tailed *p* values below 0.05 were considered statistically significant.

## 3. Results

During the considered study period, out of 421 patients who underwent CRS+HIPEC for peritoneal surface malignancies in our Institution, we selected 200 patients who fulfilled the inclusion criteria. We excluded 77 patients for high BMI, 74 with an ECOG PS > 2, 41 for incomplete records, and 29 because of age. Forty-three were male and 157 female. Mean age was 61.3 years (range 32-75). Clinical characteristics are reported in Table 1. One hundred and four patients had previous chemotherapy regimens. Twenty-eight primary advanced ovarian cancers underwent neoadjuvant treatment with a platinum/paclitaxel-based regimen. Of the remaining 76 patients, 6 had systemic chemotherapy for breast cancer several years before developing peritoneal metastases; 52 patients with peritoneal metastases from colorectal cancer had previous (from 1 to 5 years before) FOLFOX/FOLFIRI regimens. The remaining 18 patients (gastric and endometrial cancers) underwent neoadjuvant chemotherapy before cytoreduction. All patients were admitted for surgery at least two months after the end of the last chemotherapy regimen and after having proven their immune competency by blood tests. In order to achieve maximal cytoreduction, a total of 1360 surgical procedures were performed (Table 1).

An uneventful recovery was observed in 112 cases (56%). Postoperative complications were observed in 88 patients (44%). Cause of complications was infective-related in 42, noninfective-related in 37, and HIPEC-related in 9 patients. Mortality rate was 3.5%. Causes of death were sepsis and subsequent multiorgan failure in 2 patients, complications of an intra-abdominal abscess in 1 patient, and massive pulmonary embolism despite a preoperative prophylactic low molecular-weight heparin standard protocol in use in our Institution in 2 patients. The remaining 2 patients died of myocardial infarction (1) and of complications after bowel perforation (1).

Major complications (grades 3 to 4) occurred in 30 patients (15%), requiring interventional endoscopy or CT scan/ultrasound-guided procedures, surgery, or readmission in the ICU. Type and grade of the observed adverse events are reported in Table 2. Etiology of infectious complications was bacterial in 26, fungal in 6, and mixed bacterial/fungal in 10 cases. Candida spp were the most frequent species isolated, followed by E. coli, Klebsiella, and Staphylococcus epidermidis. Sixty-two positive cultures were reported in the 42 patients with infectious complications. Etiology and site of complication are reported in Table 3.

Complications were observed in 36 of 102 (35.3%) of the patients in the SGA group A and in 52 of 98 (53%) of the patients in SGA groups B/C (*p* < 0.05) while no statistical difference was observed for major complications in the 2 groups (13.7% in group A vs. 23.4% in groups B/C, *p*: ns). The infectious complication rate was higher in SGA groups B/C than in SGA group A (9.8% vs. 32.6%, *p* < 0.01).

Infectious complications occurred in 30 cases in a single site or organ, in 12 patients in multiple sites, 2 of which led to septic shock and death. Infection was the only complication in 14 patients and in 28 was associated with a surgical complication. A simultaneous infection occurred in 16 of the 58 patients (27.5%) with only one postoperative surgical complication and in 12 of the 16 patients (75%) in whom multiple postoperative surgical complications occurred (*p* < 0.003).

Univariate analysis performed to correlate possible risk factors and incidence of postoperative infective adverse events showed that ASA score, performance status, SGA, PCI, intraoperative blood loss, large bowel resection, and postoperative serum albumin level were factors significantly influencing postoperative infection rates (Table 4). Multivariate analysis (Cox regression test) confirmed the ASA score, intraoperative blood loss, large bowel resection, and SGA as independent variables significantly linked with postoperative infectious complications (Table 5).

## 4. Discussion

Mortality and morbidity rates after cytoreduction and HIPEC vary greatly in the literature [2, 5, 31]. Possible reasons could be that the definition and management themselves are subjective, often depending on the surgeon's attitude and skills [5] and that different complication classifications are currently used [11, 32]. It would be very important to standardize the adverse events reported in the literature, and the use of the NCI-CTCAE could be helpful to report adverse events in a homogeneous way to be easily compared.

Regarding type of operation, often cytoreductive surgery procedures include multiple bowel resections in the attempt to obtain a CC-0 score, and interestingly, our results identify colorectal resections as a variable influencing the postoperative infection rate. This should be carefully considered during planning of surgical strategy, avoiding large bowel resections in favor of more conservative surgical choices to reduce the risk of postoperative infectious adverse events.

Mortality (3.5%) and morbidity (44%) reported in our series match with those reported in previous studies [6, 10, 11, 17, 32, 33]. Quenet et al. recently reported the results of the PRODIGE-7 trial with a HIPEC-related 60-day major morbidity (grades 3-5) of 24.1% [34]. We observed an uneventful recovery in 56% of the patients while 88 patients experienced complications, 51 of them requiring no or only medical treatment (grade 1/2). Grade 3 to 5 adverse events, requiring interventional radiology, reoperation, or ICU readmission, were reported in 18.5% of the patients. These figures are similar to those reported for other major GI operations [25, 31].

In the few previously published studies reporting infectious complications, rates between 24 and 45% have been observed, with an infection related mortality of 1-2%. Kusamura et al. [6] reported 3.4% infectious complication rates in 209 peritonectomies followed by HIPEC, and Sugarbaker reported 42% infection rates in grade III adverse events and 5% rates in grade IV adverse events. In our series, the most common grade 1-2 infectious complications were surgical site infections and central line-associated bloodstream infections. Intra-abdominal fluid collections were the most frequent major complication (grades 3-5). Despite bacterial infections being more frequent, the Candida species were the most isolated microbial agent. This data confirms a previous report from Capone et al. [2]. Regarding nutritional status, it is crucial to emphasize that.

The importance of perioperative nutritional status in major surgery is well-known [35]. BMI has been shown to be a single clinical variable influencing surgical outcome [36–38], and the serum albumin level is a well-known factor predicting the risk of infectious complications and sepsis [39, 40], and recent papers underlined the role of immune stimulating diet or enteral nutrition prior to surgery [41]. In our study, we selected only patients within a normal BMI range to avoid subgroups, underweight or obese, with significant higher mortality and morbidity. Postoperative serum albumin levels < 2 g/dl also correlated with higher rates of infectious complications. Infectious complications occurred more frequently in the mildly (B) or severely (C) malnourished group when compared to the group of well-nourished patients (A), highlighting the upmost importance of nutritional status at the time of surgery and the need for an effective perioperative nutritional support.

In patients undergoing cytoreductive surgery and HIPEC, Uccella et al. found malnutrition as a factor predictive of a higher rate of perioperative complications in patients with advanced ovarian cancer undergoing upfront cytoreductive surgery [42], and the results of the clinical trial “Perioperative Immunonutrition for Patients Undergoing Cytoreductive Surgery (CRS) and Hyperthermic Intraperitoneal Chemotherapy” from the National Cancer Center of Singapore [43] investigating the impact of immune nutrition in reducing surgical site infections and length of hospital stay should be available in 2019. Early start of enteral or parenteral nutrition and oral feeding seems to be a protective factor towards postoperative complications, and since early postoperative enteral nutrition is not always possible in most of these patients, it has been suggested that early supplementary parenteral nutrition should be given when resuming oral feeding is estimated greater than three days [44]. Standard nutritional support (protein, vitamins, and minerals) and immunonutrition (arginine, nucleotides, and omega 3 fatty acids) seem to both be good options during the perioperative period in order to reduce the length of stay and infectious complications in cancer patients [45, 46].

## 5. Conclusions

Malnourished patients (both mild and severely) are more prone to postoperative infectious complications that account for nearly 50% of all adverse events, and the Candida species are the most frequent infective agent involved. Tumor load (PCI score), ASA score, performance and nutritional status, large bowel resection, and postoperative serum albumin levels were the variables found to significantly influence the rate of postoperative infectious complications in patients in our group of patients with peritoneal metastases undergoing CRS and HIPEC. Adequate perioperative nutritional support including immunonutrition should be considered to reduce postoperative infection rates in these patients.

## Figures and Tables

**Table 1:** Clinical characteristics.

Variables	Categories	*N*	%
ASA score	1	96	48
	2	88	44
	3	16	8

Performance status (ECOG)	0	67	33.5
	1-2	133	66.5

SGA score	A	102	51
	B	78	39
	C	20	10

Primary tumor	Ovarian	101	50.5
	Colorectal	52	26
	Gastric	16	8
	Pseudomyxoma peritonei	11	5.5
	Malignant mesothelioma	9	4.5
	Breast	6	3
	Endometrial	5	2.5

Peritoneal cancer index (PCI)	Mean (range)	16.5 (6-29)	—

Surgical procedure (tot. 1360)	Regional lymphadenectomy	198	—
	Omentectomy	186	—
	Implant excisions	164	—
	Total/subtotal parietal	162	—
	Peritonectomy		—
	Colorectal resections	138	—
	Hysterectomy and ovariectomy	118	
	Splenectomy	82	—
	Cholecystectomy	74	—
	Abdominal wall resections	50	—
	Appendectomy	48	—
	Small bowel resections	40	—
	Pelvic mass excisions	26	—
	Bladder resections	12	—
	Liver resections	10	—
	Gastric resections	4	—
	Other	48	—

Completeness of cytoreduction (CC)	CC0	134	67
	CC1	42	21
	CC2/3	24	12

**Table 2:** Postoperative adverse events (as graded by CTCAE severity score v 5.0).

Adverse events	*N*	%	Grade 1/2	Grade 3	Grade 4	Grade 5
*HIPEC-related*	**9**	**10.2**	**9**	—	—	—
Renal	6		6	—	—	—
Hematological	3		3	—	—	—
*Infectious complications*	**42**	**47.7**	**31**	**7**	**1**	**3**
Sepsis	2		—	—	—	2
Abdominal abscess	8		—	7	—	1
Central line-associated bloodstream infections	11		11	—	—	—
Wound infection	15		15	—	—	—
Pneumonia	6		5	—	1	—
*Noninfectious complications*	**37**	**42.1**	**11**	**11**	**11**	**4**
Acute postoperative pancreatitis	4		—	3	1	—
Pleural effusion	10		7	3	—	—
Deep vein thrombosis	2		2	—	—	—
Transient ischemic attack	1		—	—	1	—
Pulmonary embolism	2		—	—	—	2
Respiratory failure	4		2	—	2	—
Bowel perforation	4		—	—	3	1
Anastomotic dehiscence	1		—	—	1	—
Colostomy necrosis	1		—	—	1	—
Urinary fistula	2		—	2	—	—
Peritoneal bleeding	2		—	2	—	—
Gastric bleeding	1		—	1	—	—
Acute myocardial infarction	3		—	—	2	1
*Total*	88		51 (57.9%)	18 (20.4%)	12 (13.6%)	7 (0.8%)

**Table 3:** Site and etiology of infectious complications.

	Wound	Abdominal drain	CVL	Bloodstream	Vaginal	Urine	Total
Candida albicans	0	2	5	6	2	1	16
Escherichia coli	6	4	0	0	1	1	12
Klebsiella pneumonie	2	1	3	1	0	1	8
Enterobacter	2	4	0	0	0	0	6
Staphylococcus aureus	4	2	0	0	0	0	6
Staphylococcus coag neg	0	0	2	2	0	0	4
Enterococcus faecalis	2	2	0	0	0	0	4
Proteus mirabilis	0	0	0	0	2	0	2
Acinetobacter baumannii	0	0	1	1	0	0	2
Pseudomonas aeruginosa	2	0	0	0	0	0	2
Total	18	15	11	10	5	3	62

**Table 4:** Correlation between risk factors and infectious complications (univariate analysis).

Variables	Patients (*n* = 200) (%)	Infectious complication (*n* = 42) (%)	*p*
Age (years)			
<61	82 (41)	19 (45)	0.06
>61	118 (59)	23 (55)	
ASA score			
<3	184 (92)	12 (28)	0.018^∗^
>3	16 (8)	30 (72)	
SGA score			
A	102 (51)	11 (26)	0.016^∗^
B/C	98 (49)	31 (74)	
P. status (ECOG)			
<2	107 (53)	15 (36)	0.03^∗^
>2	93 (47)	27 (64)	
Cytoreduction			
Primary	124 (62)	20 (48)	0.1
Secondary	76 (38)	22 (52)	
PCI score			
<15	62 (31)	16 (38)	0.05^∗^
>15	138 (69)	26 (62)	
Ascites			
Yes	92 (46)	23 (55)	0.06
No	108 (54)	19 (45)	
Prev. chemotherapy			
Yes	104 (52)	24 (57)	0.07
No	96 (48)	18 (43)	
Intestinal obstruction			
Yes	42 (21)	20 (48)	0.1
No	158 (79)	22 (52)	
Colorectal resections			
Yes	138 (69)	28 (67)	0.024^∗^
No	62 (31)	14 (33)	
Length of surgical procedure (min)			
		
<423	159 (79.5)	17 (40)	0.059
>423	41 (20.5)	25 (60)	
Number of surgical procedures			
		
<4	18 (9)	18 (43)	0.07
>4	182 (91)	24 (57)	
Blood loss (ml)			
<1400	93 (46.5)	13 (31)	0.04^∗^
>1400	107 (53.5)	29 (69)	
ICU stay (hours)			
<31	78 (39)	17 (40)	0.059
>31	122 (61)	25 (60)	
HIPEC drugs			
Cisplatin	132 (66)	20 (48)	0.1
Oxaliplatin	68 (34)	22 (52)	
Post-op serum albumin (g/dl)			
		
<2	54 (27)	29 (69)	0.03^∗^
>2	146 (73)	13 (31)	

^∗^Significant.

**Table 5:** Risk factors and infectious complications: multivariate analysis (Cox regression model).

Variables	DF	Sum of squares	Mean square	*f* ratio	Prob. level
ASA score	3	2.110112	0.7033707	4.276	0.0072^∗^
<3					
>3					
Blood loss (ml)	1	0.6134862	0.6134862	3.729	0.0466^∗^
<1400					
>1400					
No. of surgical procedures	1	0.1020328	0.1020328	0.620	0.4330
<4					
>4					
Performance status	3	0.1638669	5.462231	0.332	0.8022
<2					
>2					
Colonic resections	2	0.6366483	0.8336443	3.682	0.0487^∗^
Yes					
No					
SGA score	3	0.6246453	0.6366483	2.994	0.0422^∗^
A					
B/C					
Platinum-based HIPEC	1	0.1644679	0.1664563	0.892	0.1422
Cisplatin					
Oxaliplatin					
Serum albumin levels (g/dl)	1	0.6467919	0.7366533	2.124	0.0382^∗^
<2					
>2					

^∗^Significant.

## Data Availability

The data used to support the findings of this study are available from Simone Sibio upon request.
